# Anti-cancer effects of Coix seed extract through KCTD9-mediated ubiquitination of TOP2A in lung adenocarcinoma

**DOI:** 10.1186/s13008-024-00112-2

**Published:** 2024-02-20

**Authors:** Jiuyang Jiang, Xue Li, Chun Zhang, Jiafu Wang, Jin Li

**Affiliations:** 1 Department of Thoracic Surgery, The First Affiliated Hospital of Harbin Medical University, Harbin, 150001 Heilongjiang People’s Republic of China; 2 Department of Internal Medicine, Daoli District People’s Hospital, Harbin, 150016 Heilongjiang People’s Republic of China; 3 Department of Traditional Chinese Medicine, The First Affiliated Hospital of Harbin Medical University, Harbin, 150001 Heilongjiang People’s Republic of China; 4 Department of PET-CT, The First Affiliated Hospital of Harbin Medical University, Harbin, 150001 Heilongjiang People’s Republic of China; 5 Department of Traditional Chinese Medicine, The Fourth Affiliated Hospital of Harbin Medical University Songbei, No. 766, Xiang’an North Street, Songbei District, Harbin, 150070 Heilongjiang People’s Republic of China

**Keywords:** Lung adenocarcinoma, Coix seed extract, KCTD9, TOP2A, Ubiquitination modification

## Abstract

**Background:**

Coix seed extract (CSE), a traditional Chinese medicine, has been reported as an adjunctive therapy in cancers. However, the molecular targets are largely unclear. The study is designed to unveil its function in lung adenocarcinoma (LUAD) and the possible molecular mechanism.

**Methods:**

The HERB database was utilized to predict the molecular targets of the Coix seed, followed by prognostic value prediction in the Kaplan–Meier Plotter database. LUAD cells were infected with sh-KCTD9 after co-culture with CSE, and cell viability, growth, proliferation, and apoptosis were determined. The substrates of KCTD9 were predicted using a protein–protein interaction network and verified. The expression of PD-L1, the contents of TNF-α, IFN-γ, CXCL10, and CXCL9 in the co-culture system of LUAD cells and T cells and the proliferation of T cells were evaluated to study the immune escape of LUAD cells in response to CSE and sh-KCTD9. Lastly, tumor growth and immune escape were observed in tumor-bearing mice.

**Results:**

CSE inhibited malignant behavior and immune escape of LUAD cells, and the reduction of KCTD9 reversed the inhibitory effect of CSE on malignant behavior and immune escape of LUAD cells. Knockdown of KCTD9 expression inhibited ubiquitination modification of TOP2A, and knockdown of TOP2A suppressed immune escape of LUAD cells in the presence of knockdown of KCTD9. CSE exerted anticancer effects in mice, but the reduction of KCTD9 partially compromised the anticancer effect of CSE.

**Conclusion:**

CSE inhibits immune escape and malignant progression of LUAD through KCTD9-mediated ubiquitination modification of TOP2A.

## Background

The incidence of lung cancer at advanced disease continued to decline while rates for localized-stage increased by 4.5% annually, leading to gains in the proportion of localized-stage diagnoses and 3-year survival (from 21 to 31%) [1]. Lung cancer is classified as small cell lung cancer or non-small cell lung cancer (NSCLC) for treatment, of which lung adenocarcinoma (LUAD) and lung squamous cell carcinoma (LUSC) are the most frequent subgroups [2]. Even though immunotherapy has brought more and more good news to lung cancer patients, the role of traditional Chinese medicine in the control of treatment-related symptoms is becoming more prominent [3].

Back in 2003, the methanolic extract of Coix seed was found to exert an antiproliferative effect on A549 lung cancer cells by inducing cell cycle arrest and apoptosis and suppressing tumor growth in vivo in a dose-dependent manner [4]. After that, Kanglaite (KLT), extracted from Coix seed, has been reported to produce an obvious time and dose-dependent inhibitory effect on various cancer cells, including triple-negative breast cancer [5], hepatocellular carcinoma [6], as well as NSCLC [7]. Even though it could inhibit some anti-apoptotic genes and activate some pro-apoptotic genes [8], the novel targets of Coix seed extract (CSE) in LUAD need to be further explored. Here, we identified potassium (K+) channel tetramerization domain (KCTD)9 as a target of CSE that is linked to satisfactory outcomes for patients with lung cancer in the bioinformatics prediction website. The human family of KCTD proteins counts 25 members, and many of them are only partially characterized in tumorigenesis [9]. Interestingly, the mRNA and protein expression of KCTD9 was significantly decreased in LUAD and was positively correlated with the infiltration level of macrophages and CD8^+^ T cells in the tumor tissues (Rho greater than 0.1 and *p-value* less than 0.05) [10].

A high clonal neoantigen burden in NSCLC is associated with an inflamed tumor microenvironment, enriched with activated effector T cells and the expression of proteins associated with T cell migration (CXCL10 and CXCL9), as well as negative regulators of T-cell activity including PD-L1 [11]. Since immune checkpoint blockade with PD-L1 antibodies can generate durable responses in patients with advanced NSCLC [12], the immune escape represents one of the major hurdles to be overcome to improve clinical outcomes in patients treated with immunotherapy [13]. Therefore, dissecting the possible role played by KCTD9 in CD8^+^ T cell activity and the immune escape might provide further evidence supporting the antitumor effects of CSE in LUAD.

## Results

### CSE suppresses the malignant aggressiveness of LUAD cells

**Fig. 1:**
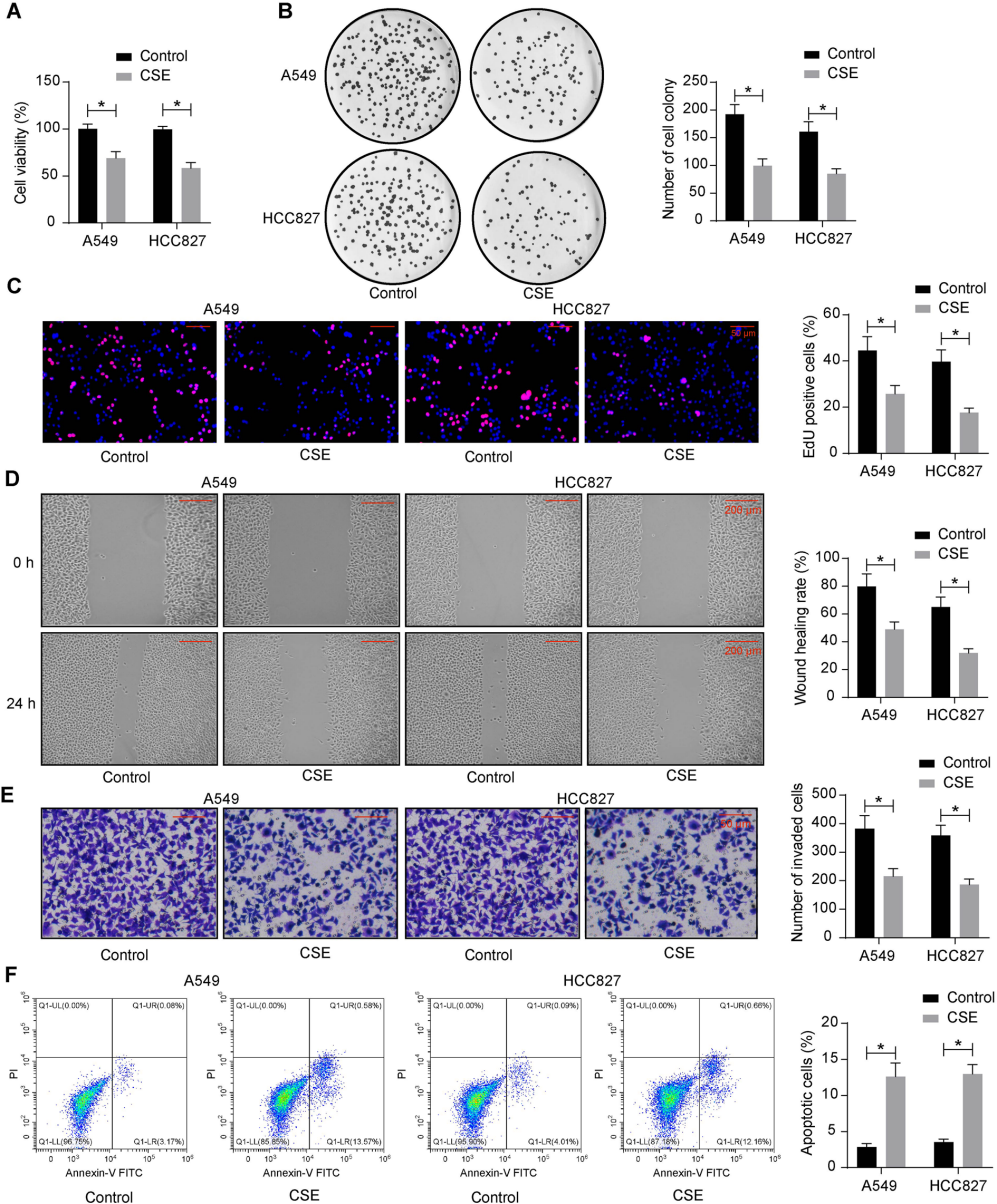
CSE exerts an anti-tumor effect on LUAD cells. **a** LUAD cell viability was examined using CCK-8. **b** LUAD cell growth was measured using colony formation assays. **c** LUAD cell proliferation was measured using EdU assays. **d** LUAD cell migration was measured using wound healing assays. **e** LUAD cell invasion was measured using the Transwell invasion assay. **f** Detection of apoptosis in LUAD cells by flow cytometry. Data are presented as the mean ± SD of results from at least three independent experiments performed in triplicates, two-way ANOVA with Tukey’s posttests, *p < 0.05

The effects of CSE on the cell viability of A549 and HCC827 cells were analyzed by CCK-8 assays. The cell viability of the cells in the CSE group was decreased compared with that in the control group (Fig. 1A). Colony formation and EdU assays showed a decrease in colony formation and EdU-positive cells in the CSE group of cells compared to the control group (Fig. 1B, C). The effect of CSE on cell migration was analyzed using wound healing assays. In the control group, the wounds were almost healed after 24 h; whereas in the CSE group cells, the wound healing rate was significantly decreased (Fig. 1D). The cell invasion was observed by the Transwell invasion assay, which demonstrated that the number of CSE-treated cells invading through Matrigel was reduced (Fig. 1E). The apoptosis was detected by flow cytometry. The rate of apoptosis in the CSE group was significantly higher than that in the control group, suggesting that CSE could induce apoptosis in LUAD cells (Fig. 1F).

### Reduction of KCTD9 mitigates the inhibitory effect of CSE on the malignant behavior of LUAD cells

**Fig. 2:**
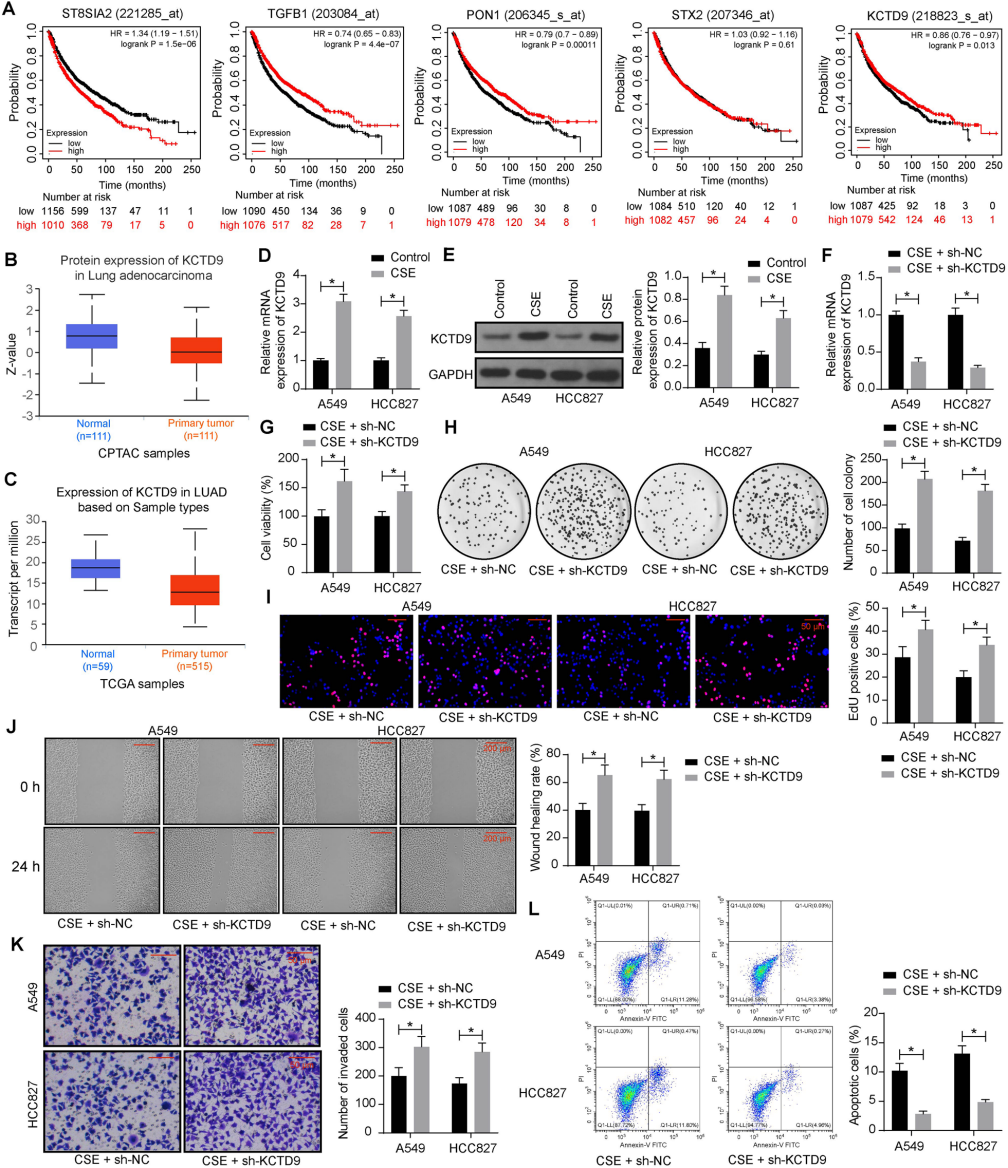
Reduction of KCTD9 mitigates the inhibitory effect of CSE on malignant behavior in LUAD cells. **a** The correlation between patient prognosis’ and PON1, TGFB1, STX2, ST8SIA2, and KCTD9 in lung cancer in the Kaplan–Meier Plotter database. **b** The protein expression of KCTD9 in LUAD in the UALCAN database. **c** The mRNA expression of KCTD9 in LUAD in the UALCAN database. **d** Effect of CSE on KCTD9 mRNA expression in LUAD cells by RT-qPCR. **e** Effect of CSE on KCTD9 protein expression in LUAD cells by western blot analysis. LUAD cells were infected with sh-NC or sh-KCTD9 and treated with CSE. **f** Effect of sh-NC or sh-KCTD9 on KCTD9 mRNA expression in LUAD cells by RT-qPCR. **g** LUAD cell viability was examined using CCK-8. **h** LUAD cell growth was measured using a colony formation assay. **i** LUAD cell proliferation measured using EdU assay. **j** LUAD cell migration measured using wound healing assay. **k** LUAD cell invasion measured using Transwell invasion assay. **l** Detection of apoptosis in LUAD cells by flow cytometry. Data are presented as the mean ± SD of results from at least three independent experiments performed in triplicates, two-way ANOVA with Tukey’s posttest, *p < 0.05

We predicted the molecular targets for the Coix seed in the Chinese medicine database HERB (http://herb.ac.cn/) which gathered 472 high-throughput GEO datasets containing 6,164 GEO samples and ordered them from smallest to largest according to the *p*-value. We analyzed the prognostic value of the top five genes PON1, TGFB1, STX2, ST8SIA2, and KCTD9 in lung cancer in the Kaplan–Meier Plotter database (http://kmplot.com/analysis/index.php?p=background). The results showed a correlation between PON1, TGFB1, ST8SIA2, and KCTD9 and the prognosis of patients (Fig. 2A). In LUAD, mRNA and protein expression of KCTD9 was reported to be significantly reduced [10]. Consistently, in the UALCAN database (https://ualcan.path.uab.edu/index.html), we also found that the protein and mRNA expression of KCTD9 was significantly reduced in LUAD (Fig. 2B, C). RT-qPCR and western blot assays were utilized to detect the KCTD9 mRNA and protein expression in the LUAD cells with or without CSE treatment. KCTD9 expression was significantly enhanced in the CSE group compared to the control group (Fig. 2D, E), which indicated that CSE promoted the mRNA and protein expression of KCTD9.

To examine the involvement of KCTD9 in the anti-tumor effects of CSE, we delivered Lentivirus-encapsulated KCTD9 knockdown plasmid into LUAD cells, followed by CSE treatment. RT-qPCR assay results verified the successful downregulation of KCTD9 in cells in the CSE + sh-KCTD9 group (Fig. 2F). The cell viability and colony formation were significantly increased following KCTD9 knockdown (Fig. 2G, H). EdU assay showed that there was a significant elevation of cell growth in the CSE + sh-KCTD9 group (Fig. 2I). Moreover, KCTD9 knockdown contributed to enhanced migration and invasion even after CSE treatment, as revealed by wound healing and Transwell invasion assays (Fig. 2J, K). Flow cytometry results showed that the cells infected with sh-KCTD9 showed elevated apoptosis resistance (Fig. 2L).

### CSE weakened the immune evasion of LUAD cells by promoting KCTD9

**Fig. 3:**
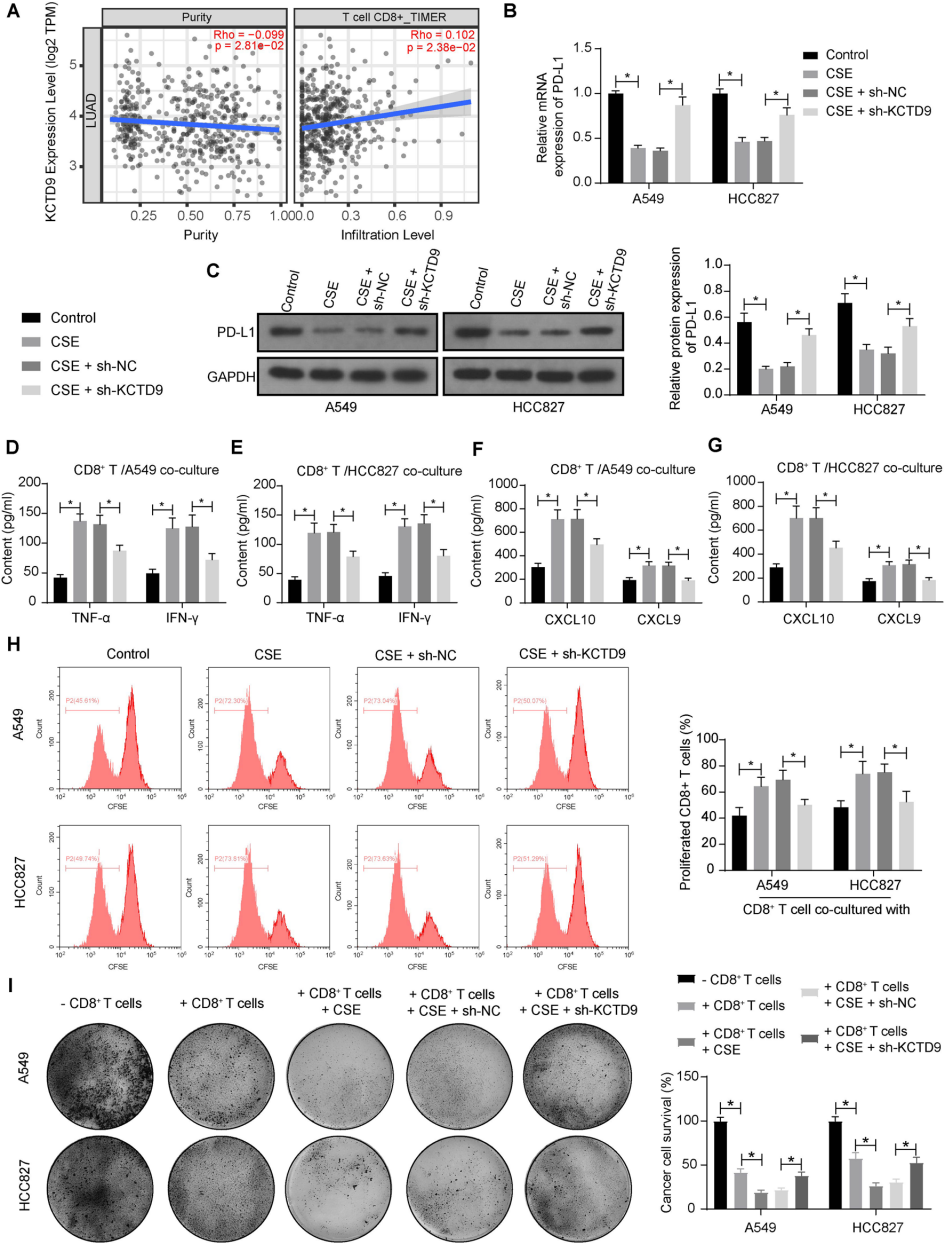
The inhibiting effects of CSE on the immune escape of LUAD cells are reversed by KCTD9 knockdown. **a** Correlation between KCTD9 and T-cell infiltration in LUAD analyzed by TIMER 2.0 database. **b** PD-L1 mRNA expression in LUAD cells with different treatments detected by RT-qPCR assays. **c** PD-L1 protein expression in LUAD cells with different treatments detected by western blot assays. **d**-**g** The levels of TNF-α, IFN-γ, CXCL10, and CXCL9 in the co-culture system of LUAD cells and human CD8^+^ T cells. **h** Proliferation of human CD8^+^ T cells in co-culture system detected by CFSE assays. **i** The ability of differently treated LUAD cells against human CD8^+^ T cells was evaluated using tumor cell killing assay. Data are presented as the mean ± SD of results from at least three independent experiments performed in triplicates, two-way ANOVA with Tukey’s posttest, *p < 0.05

We analyzed the correlation between KCTD9 and T-cell infiltration in LUAD in the TIMER 2.0 database (http://timer.cistrome.org/). The expression of KCTD9 was significantly and positively correlated with CD8^+^ T cell infiltration (Fig. 3A). To investigate the effects of CSE and KCTD9 on immune escape in LUAD, we examined the expression of PD-L1 in A549 and HCC827 cells. The results of RT-qPCR and western blot analyses showed that LUAD cells after CSE treatment had lower expression of PD-L1 than that in the control group. By contrast, the expression of PD-L1 was increased following sh-KCTD9 infection in LUAD cells relative to cells infected with sh-NC (Fig. 3B, C). We also detected the levels of TNF-α, IFN-γ, CXCL10, and CXCL9 in the human CD8^+^ T cell/LUAD cell co-culture system. Higher levels of TNF-α, IFN-γ, CXCL10, and CXCL9 were observed in the co-culture system consisting of LUAD cells treated with CSE, whereas the levels of these cytokines and chemokines were reduced in response to KCTD9 knockdown (Fig. 3D–G). We further explored the effects of CSE and KCTD9 on the proliferation of human CD8^+^ T cells in the co-culture system. The CFSE assay showed that the number of human CD8^+^ T cells co-cultured with CSE-treated cells was increased, while KCTD9 knockdown reduced the number of proliferative human CD8^+^ T cells (Fig. 3H). We further found that LUAD cell survival was significantly downregulated after the addition of CD8^+^ T cells, as analyzed by a tumor cell killing assay; thereafter CSE treatment further suppressed the LUAD cell survival. By contrast, the combined knockdown of KCTD9 reversed the inhibitory effect of CSE on the survival of cancer cells (Fig. 3I).

### KCTD9 affects the level of ubiquitination of TOP2A

**Fig. 4:**
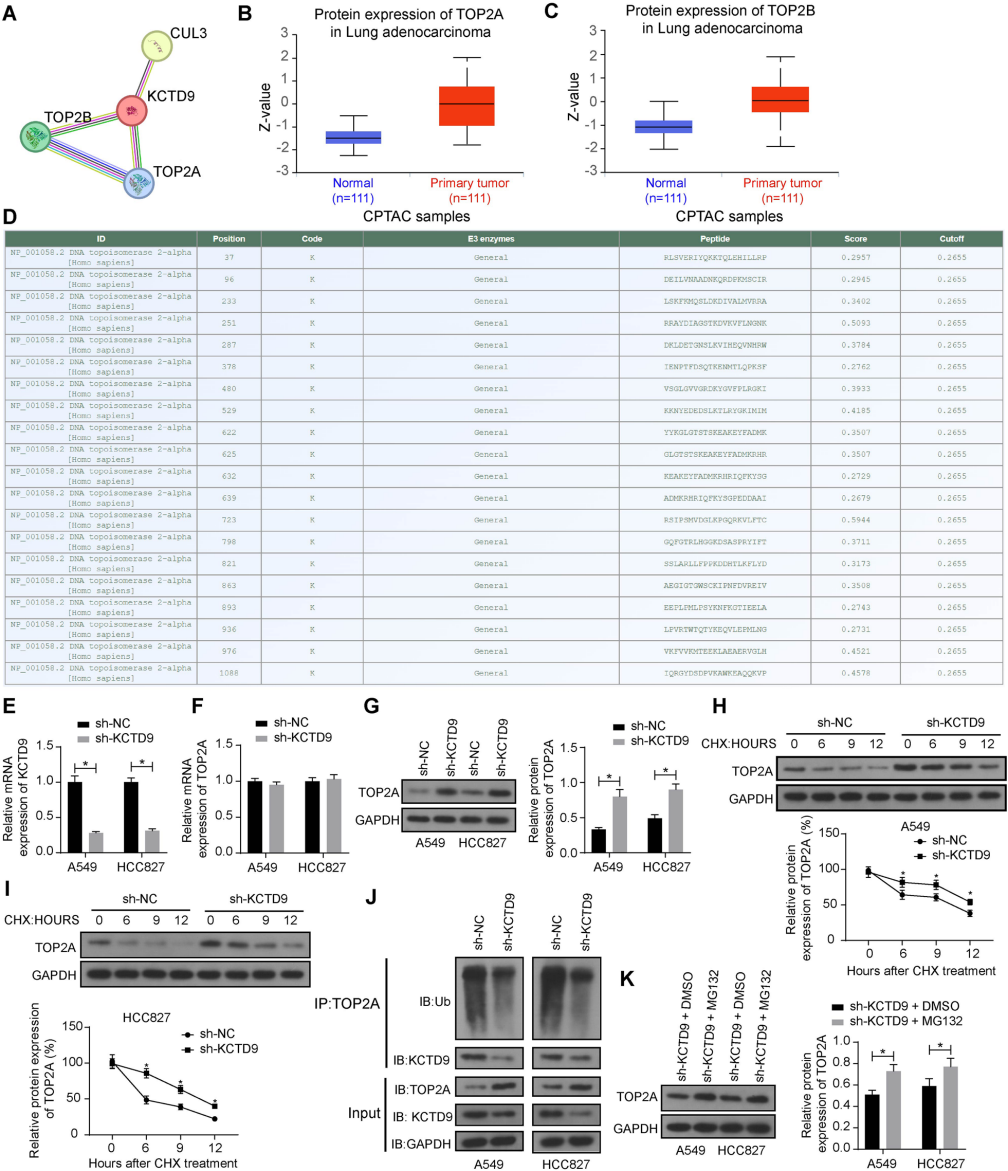
KCTD9 affects the level of ubiquitination of TOP2A. **a** Interactions between TOP2A and TOP2B and KCTD9 in the String database. Protein expression of TOP2A (**b**) and TOP2B (**c**) in LUAD was analyzed in the UALCAN database. **d** Multiple ubiquitination modification sites are present in TOP2A in the GPS-Uber database. **e** The mRNA expression of KCTD9 in LUAD cells after infection of sh-NC or sh-KCTD9 was verified using RT-qPCR. **f** The mRNA expression of TOP2A in LUAD cells after infection of sh-NC or sh-KCTD9 was verified using RT-qPCR assays. **g** The protein expression of TOP2A in LUAD cells after infection of sh-NC or sh-KCTD9 was verified using western blot assays. The expression of TOP2A in A549 (**h**) and HCC827 (**i**) cells after infection of sh-NC or sh-KCTD9 in the presence of CHX. **j** Effect of KCTD9 on TOP2A ubiquitination detected by immunoprecipitation. **k** The protein expression of TOP2A in LUAD cells after infection of sh-NC or sh-KCTD9 with the proteasome inhibitor MG132 treatment. Data are presented as the mean ± SD of results from at least three independent experiments performed in triplicates, two-way ANOVA with Tukey’s posttest, *p < 0.05

We analyzed the protein–protein interaction (PPI) network of KCTD9 in the String database (https://version-12-0.string-db.org/cgi/input?sessionId=bt2mfBvNOXsP&input_page_show_search=off). Setting high confidence, we noticed the interactions between TOP2A, TOP2B, Cullin3 (Cul3), and KCTD9 (Fig. 4A). Further analyzing the protein expression of TOP2A and TOP2B in LUAD in the UALCAN database, we found that both were increased in LUAD (Fig. 4B, C), but the significance of TOP2A (8.41225406623592E-22) was higher than TOP2B (3.17397112747271E-18). In the GPS-Uber database (http://gpsuber.biocuckoo.cn/index.php), based on the protein sequence of TOP2A, we found multiple ubiquitination modification sites (Fig. 4D). However, it is unknown whether KCTD9 inhibits LUAD progression by inducing its degradation through ubiquitination modification.

To investigate the effect of KCTD9 on the level of TOP2A ubiquitination in LUAD cells, we reduced the expression of KCTD9 in A549 and HCC827 cells in the absence of CSE. After verifying the successful knockdown of KCTD9 in the A549 and HCC827 cells using RT-qPCR (Fig. 4E), we conducted RT-qPCR and western blot assays to determine the mRNA and protein expression of TOP2A. Even though the mRNA expression of TOP2A was not significantly changed (Fig. 4F), the expression of TOP2A was upregulated in the A549 and HCC827 cells infected with sh-KCTD9 (Fig. 4G).

To determine whether KCTD9 regulates TOP2A stability, we examined the protein levels of TOP2A in the presence of CHX and KCTD9 knockdown. Western blot results demonstrated that knocking down KCTD9 promoted the stability of the TOP2A protein (Fig. 4H, I). Next, we tested whether the ubiquitination of TOP2A was affected by KCTD9. Knocking down the expression of KCTD9 decreased the ubiquitination level of TOP2A (Fig. 4J). Then, we treated cells in the sh-KCTD9 groups with MG132 to compare the changes in TOP2A protein expression. An increase in the TOP2A protein expression was observed following MG132 treatment (Fig. 4K).

### Silencing of TOP2A hampers the immune escape of LUAD cells

**Fig. 5:**
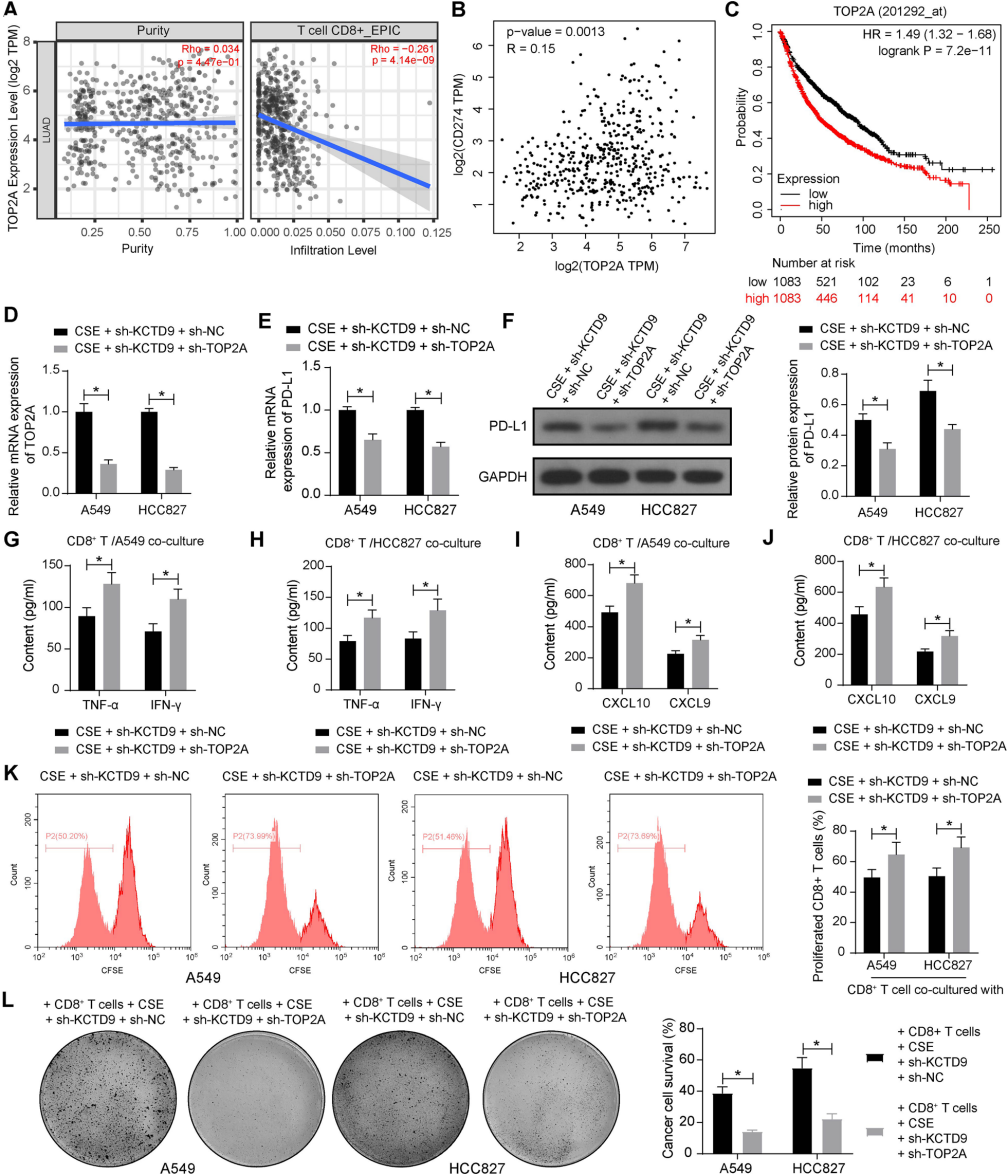
Silencing of TOP2A reverses the effects of KCTD9 knockdown on LUAD cell immune escape. **a** Correlation of TOP2A expression in LUAD with human CD8^+^ T cell infiltration predicted in the TIMER database. **b** Correlation between TOP2A and PD-L1 expression predicted in the GEPIA database. **c** The prognostic outcomes in patients with high or low TOP2A expression are predicted in the Kaplan–Meier Plotter database. **d** The mRNA expression of TOP2A in LUAD cells after infection of sh-NC or sh-TOP2A was determined using RT-qPCR. **e** The mRNA expression of PD-L1 in LUAD cells was detected using RT-qPCR. **f** The protein expression of PD-L1 in LUAD cells was detected using western blot assays. **g**-**j** The levels of TNF-α, IFN-γ, CXCL10, and CXCL9 in the co-culture system of LUAD cells and human CD8^+^ T cells. **k** Proliferation of human CD8^+^ T cells in co-culture system detected by CFSE assays. **l** The ability of differently treated LUAD cells against human CD8^+^ T cells was evaluated using tumor cell killing assay. Data are presented as the mean ± SD of results from at least three independent experiments performed in triplicates, two-way ANOVA with Tukey’s posttest, *p < 0.05

The TIMER database revealed that TOP2A expression was negatively correlated with CD8^+^ T cell infiltration in LUAD (Fig. 5A), and there is a significant positive correlation between TOP2A and PD-L1 in the GEPIA database (http://gepia.cancer-pku.cn/detail.php) (Fig. 5B). The Kaplan–Meier Plotter database showed that patients with low expression of TOP2A had a significantly better prognosis (Fig. 5C).

To characterize the role of TOP2A in the immune escape of LUAD, we also knocked down the expression of TOP2A (sh-NC as control) along with sh-KCTD9 and CSE treatment. The successful knockdown of TOP2A (Fig. 5D) led to decreased PD-L1 mRNA and protein expression in the infected cells (Fig. 5E, F). The two groups of cells were co-cultured with human CD8^+^ T cells, and ELISA kits were used to detect the content of cytokines and chemokines. The contents of TNF-α, IFN-γ, CXCL10, and CXCL9 were elevated in the co-culture system consisting of LUAD cells with sh-TOP2A (Fig. 5G–J). CFSE assay to detect the proliferation of human CD8^+^ T cells in the co-culture system showed that the proliferative number of human CD8^+^ T cells was elevated after TOP2A knockdown (Fig. 5K). In addition, it was found by tumor cell killing assay that the knockdown of TOP2A significantly downregulated the viability of LUAD cells (Fig. 5L).

### CSE weakened the immune evasion of LUAD cells in vivo by promoting KCTD9

**Fig. 6:**
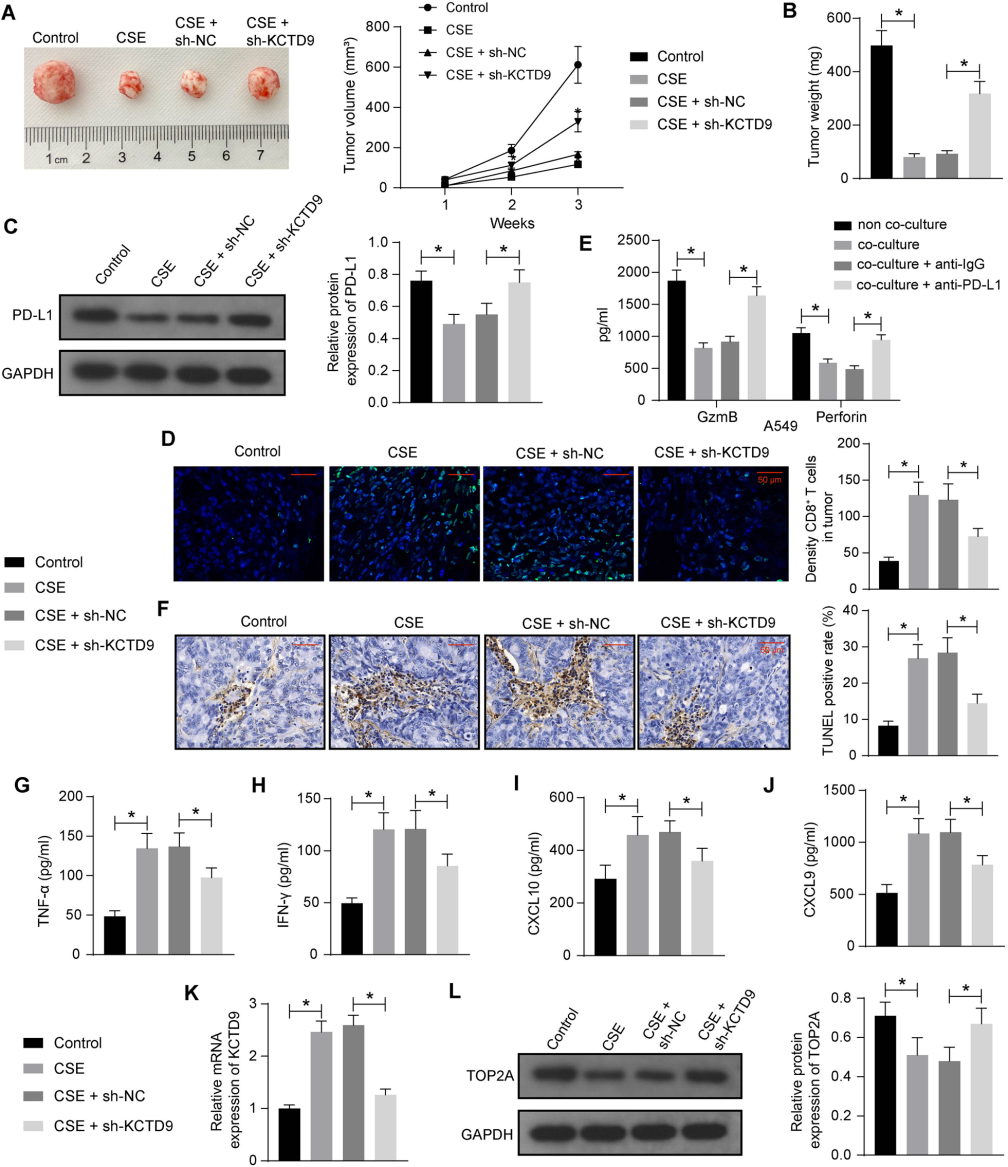
The inhibiting effects of CSE on the immune escape of LUAD cells in vivo are reversed by KCTD9 knockdown. **a** Tumor volume in mice in 3 weeks. **b** Weight changes in mouse tumors. **c** The protein expression of PD-L1 in mouse tumor tissues was examined using western blot assays. **d** Immunofluorescence analysis of CD8^+^ T cell infiltration in mouse tumor tissues. **e** The levels of CD8^+^ T cell activation markers GzmB and Perforin in the supernatant of the co-culture system were detected by ELISA. **f** Detection of apoptosis in mouse tumor tissues by TUNEL assay. The levels of TNF-α (**g**), IFN-γ (**h**), CXCL10 (**i**), and CXCL9 (**j**) in the mouse tumor tissues by ELISA. (**k**) The mRNA expression of KCTD9 in mouse tumor tissues was assessed by RT-qPCR. **l** The protein expression of TOP2A in mouse tumor tissues was assessed by western blot assays. Data are presented as the mean ± SD of results from at least three independent experiments performed in triplicates, one-way or two-way ANOVA with Tukey’s posttest, *p < 0.05

For the in vivo experiments, we measured the tumor volume of mice in the control, CSE, CSE + sh-NC, and CSE + sh-KCTD9 groups with calipers, after which the tumors were isolated and weighed. The tumor volume and weights of the mice administrated with CSE were smaller than that of the control mice, and the tumor volume and weight of mice in the CSE + sh-KCTD9 group were larger and heavier than those of mice injected with CSE + sh-NC (Fig. 6A, B). The expression of PD-L1 in the tumor tissues of mice was detected by western blot analysis. There was a significant decline in the expression of PD-L1 in the tumor tissues of CSE-treated mice and an elevation in the expression of PD-L1 in the tumor tissues of mice administrated with CSE + sh-KCTD9 (Fig. 6C). Then, CD8^+^ T cell infiltration in tumor tissues of mice was analyzed using immunofluorescence staining. The results showed that the density of CD8^+^ T cells in tumor tissues of CSE mice was increased. The density of CD8^+^ T cells in tumor tissues of sh-KCTD9-treated mice was, however, decreased (Fig. 6D).

Then we co-cultured CD8^+^ T cells isolated from mouse spleens with A549 cells at a ratio of 3:1 in vitro and blocked the expression of PD-L1 in cancer cells by PD-L1-neutralizing antibody. ELISA revealed that the expression of the mouse CD8^+^ T cell activation markers, GzmB and Perforin, was significantly suppressed by the co-culture with A549 cells. However, the expression of GzmB and Perforin was significantly upregulated in mouse CD8^+^ T cells after being treated with PD-L1 neutralizing antibody (Fig. 6E). This suggests that PD-L1 on the surface of human LUAD cells can inhibit CD8^+^ T cell activation by binding to PD-1 on the surface of mouse CD8^+^ T cells, thereby promoting the immune escape of LUAD cells.

Consistent with our in vitro evidence, CSE was found to play a pro-apoptotic effect in the tumor tissues of mice, which was reversed by the KCTD9 knockdown (Fig. 6F). Moreover, the toxic substances released by CD8^+^ T cells into the tumor tissues were enhanced by CSE and lowered by sh-KCTD9 (Fig. 6G–J). Lastly, KCTD9 silencing was effective in the tumor tissues of mice injected with sh-KCTD9 (Fig. 6K), and downregulation of KCTD9 led to the restoration of TOP2A protein expression (Fig. 6L).

## Discussion

Coix seed oil has been reported to stimulate immune function and promote tumor cell apoptosis of various cancer cells [14]. In the current study, we provide evidence that CSE is involved in regulating phenotypic modulation as well as immune evasion in LUAD in vitro and in vivo. Furthermore, our data suggest that CSE may regulate the ubiquitination modification of TOP2A by affecting the expression of KCTD9 in LUAD cells.

Coix seed is the seed of the perennial herbaceous plant coix and has drawn wide attention due to its beneficial effects on health [15]. For instance, CSE could augment the efficacy of gemcitabine therapy in pancreatic cancer cells [16]. According to a recent meta-analysis, CSE is effective and safe as an adjunctive treatment for NSCLC [17]. CSE has also been indicated to significantly inhibit the growth of MDA-MB-231 xenografts in athymic nude mice by downregulating COX-2 and matrix metalloproteinases which are related to cancer invasion and metastasis [18]. In the present study, the CSE treatment repressed LUAD cell viability and mobility. Subsequently, we identified KCTD9 as a target of CSE that is responsible for the antitumor effects of CSE and linked the immunoregulatory role of CSE to KCTD9 restoration in LUAD. Colorectal cancer patients whose tumors expressed low KCTD9 levels had unsatisfactory outcomes, and KCTD9 depletion induced cell proliferation and metastasis [19]. However, there is lacking information regarding the functional role of KCTD9 in LUAD. Here, we found that the silencing of KCTD9 overturned the antitumor effects of CSE on LUAD cells. Molecular docking demonstrated the strong binding relation between the main active compounds of KLT injection (Isoarborinol and Sitosterol alpha1) and the targets (CDK1, CDK2, and CHEK1) in triple-negative breast cancer [5]. Therefore, molecular docking is needed to further determine the binding relation between CSE and KCTD9.

CD8^+^ cytotoxic T cells represent a subtype of effector T cells that induce protective immunity in response to tumors, and the immunomodulatory effects of traditional Chinese medicine on immune cells and molecules have highlighted their application as immune checkpoint modulators in cancers [20]. Since KCTD9 has shown a positive correlation with the infiltration of CD8^+^ T cells, we set to decipher whether CSE can hamper immune evasion in NSCLC by restoring KCTD9 expression. High plasma levels of CXCL9 and CXCL10 were associated with better response and prolonged progression-free survival in patients with NSCLC [21]. In this research, we found that the killing effects (evidenced by TNF-α, IFN-γ, CXCL9, and CXCL10 levels) and proliferation of human CD8^+^ T cells were strengthened by CSE treatment and weakened by KCTD9 knockdown, indicating that the immunomodulatory effects of CSE on LUAD were elicited through KCTD9. Pien Tze Huang, a valuable traditional Chinese medicine, was also found to suppress the immune escape of colorectal cancer and elevated infiltration of CD8^+^ T cells in tumor tissues, which depends on the suppression of PD-L1 levels [22].

It was well-established that the KCTD family are soluble non-channel proteins that generally serve as Cul3-dependent E3 ligases [23]. Therefore, we hypothesized that KCTD9 has a substrate to play a tumor-suppressive role in LUAD. As revealed by the PPI network, TOP2A and TOP2B show a close interaction with KCTD9 in addition to Cul3. Since TOP2A expression has a more pronounced elevation in LUAD, it was chosen as the target of KCTD9. PTEN plays a role in modulating TOP2A protein stability by supporting OTUD3-catalyzed TOP2A deubiquitination [24]. We also validated the ubiquitination modification of TOP2A by KCTD9 in LUAD cells. As for its role in cancer, TOP2A expression showed an aberrant gain in NSCLC tissues compared to normal tissues, and TOP2A was associated with worse NSCLC patients’ survival [25, 26]. Knockdown of TOP2A in LUAD cells repressed cell proliferation, migration, and invasion [27]. More importantly, Nikanjam et al*.* showed that TOP2A positivity was associated with PD-L1 expression in NSCLC (*p* < 0.001) [28]. TOP2A has been recently revealed to play an important role in immunotherapy and vasculogenic mimicry formation in NSCLC through the upregulation of PD‑L1 expression [29]. In the present study, we revealed that the knockdown of TOP2A again overturned the impact of sh-KCTD9 on the CD8^+^ cells and PD-L1 expression. Epidermal growth factor receptor (EGFR) mutation is the most common driver of NSCLC [30]. HCC827 cells, one cell line used in this study, has an acquired mutation in the EGFR tyrosine kinase domain (E746–A750 deletion) [31], which might explain the higher expression of baseline PD-L1 expression in HCC827 cells than A549 cells and consistent findings were observed in vivo as well by Rios-Doria et al*.* [32].

## Conclusion

In summary, CSE repressed immune escape and malignant progression in LUAD, and the mechanism was related to KCTD9-mediated TOP2A ubiquitination. As the overall survival rate of LUAD still needs to be improved, the current discovery could be a promising strategy for LUAD treatment.

## Materials and methods

### CSE preparation

The dried powder of Coix seed (C160203, Herb Green Health Biotech Co., Ltd., Ganzhou, Jiangxi, China) was extracted by refluxing with 95% ethanol for 2 h and concentrated by mixing in a rotary evaporator. It was then dried in a vacuum drying oven at 80 °C to obtain CSE, and the CSE was stored frozen at − 4 °C. The active ingredients of CSE are coix seed fat, protein, and carbohydrates. CSE had a polysaccharide content greater than 30% [33].

### Cell lines and culture

Human LUAD cells A549 (CL-0016) and HCC827 (CL-0094, both from Procell, Wuhan, Hubei, China) were categorized into ten groups: the control, CSE, CSE + short hairpin RNA (sh)-negative control (NC), CSE + sh-KCTD9, sh-NC, sh-KCTD9, sh-KCTD9 + dimethylsulfoxide (DMSO), sh-KCTD9 + MG132, CSE + sh-KCTD9 + sh-NC, and CSE + sh-KCTD9 + sh-TOP2A groups. A549 cells were cultured in the A549 cell-specific medium (CM-0016, Procell) containing Ham's F-12 K + 10% fetal bovine serum (FBS) + 1% penicillin/streptomycin, while HCC827 cells were cultured in the HCC827 cell-specific medium (CM-0094, Procell) containing RPMI-1640 + 10% FBS + 1% penicillin/streptomycin. Subsequently, the cells were incubated in a 5% CO_2_ incubator at 37 °C. Both cell lines were identified by short tandem repeat and maintained normal and healthy cell morphology. They were free of mycoplasma contamination throughout the experiment.

### Plasmid or lentiviral construction and cell treatment

Lentivirus-encapsulated KCTD9 or TOP2A knockdown plasmid was used to infect the cells to achieve the downregulation of KCTD9 or TOP2A. Stably transfected cells were selected with 10 μg/mL puromycin 24 h after cell infection with lentivirus. For CSE treatment, the cells were treated with 300 μg/mL of CSE for 72 h, and those treated with the same dose of DMSO were set as the control group.

Cells in the sh-NC group and the sh-KCTD9 group were treated with 25 μg/mL of cycloheximide (CHX, HY-12320, MedChemExpress, Monmouth Junction, NJ, USA), and changes in cellular TOP2A expression were detected by western blot assays at four-time points, namely, 0 h, 6 h, 9 h and 12 h after treatment. For MG-132 treatment, the cells were reacted with 100 μM MG-132 (S1748, Beyotime Biotechnology Co., Ltd., Shanghai, China) for 12 h.

### Isolation of human and mouse CD8^+^ T cells

Human CD8^+^ T cells were isolated from healthy donor peripheral blood mononuclear cells (PBMCs) using the Dynabeads Untouched Human CD8 T Cell Kit (11348D, Thermo Fisher Scientific Inc., Waltham, MA, USA). An anti-non-CD8 cell antibody mixture was added to the PBMCs to enable them to bind to the cells. Dynabeads™ was added to bind to antibody-labeled cells in a short incubation. Microbead-bound cells on the magnetic rack were rapidly isolated and discarded. The remaining negatively isolated contaminant-free human CD8^+^ T cells were used for co-culture with LUAD cells [34].

Splenocytes were isolated from the spleens of C57BL/6N mice (Beijing Vital River Laboratory Animal Technology Co., Ltd., Beijing, China). CD8^+^ T cells were screened from mouse splenocytes using CD8 (TIL) MicroBeads, mouse (130-116-478, Miltenyi Biotec Inc., Auburn, CA, USA) according to the manufacturer’s instructions.

### Cell co-culture

LUAD cells and CD8^+^ T cells were co-cultured using Transwell co-culture plates (0.4 μm wells). CD8^+^ T cells were seeded into the apical chamber, while LUAD cells after different treatments were placed in the basolateral chamber. The cells were co-cultured by adding 10% FBS and 5% CO_2_ at 37 °C. After 24 h, the cell-free supernatant from the co-culture system was collected for ELISA, and CD8^+^ T cells were collected to detect their proliferation.

### CCK-8 and colony formation assays

A549 and HCC827 cells were seeded in 96-well plates (5 × 10^3^ cells/well) and incubated at 37 °C for 24 h. Cell viability was assayed using the CCK-8 kit (C0037, Beyotime), and the optical density (OD) value was measured at 450 nm.

A549 and HCC827 cells were plated into 6-well plates (500 cells/well) and cultured for 10 days, and then the cells were fixed with 4% paraformaldehyde and stained with crystal violet staining solution (C0121-100 ml, Beyotime). Colonies were counted under a light microscope and analyzed by Image J software.

### Cell proliferation assays

Cell proliferation was detected with the BeyoClick™ EdU-555 Cell Proliferation Assay Kit (C0075S, Beyotime). The cells were incubated in 6-well plates, and EdU working solution (20 μM) pre-warmed at 37 °C was added to the 6-well plates for a 1-h incubation. After the EdU labeling of cells was completed, the culture medium was removed. The cells were fixed by adding 1 mL of 4% paraformaldehyde for 15 min, incubated with phosphate-buffered saline (PBS) permeabilization solution for 10–15 min, and with 0.5 mL of Click reaction solution for 30 min in the dark (all at room temperature). Cell nuclei were stained with Hoechst 33,342 staining solution (C1025, Beyotime), and then cell proliferation was observed under a fluorescence microscope.

CD8^+^ T cell proliferation in the co-culture system was assayed with the CellTrace™ CFSE Cell Proliferation Kit (C34554, Thermo Fisher), and CFSE density was measured by flow cytometry.

### Wound healing assay and Transwell invasion assay

Cells were cultured in 6-well plates (5 × 10^3^ cells/well). When the cells reached 80–90% confluence, a sterilized 10-μl pipette tip was used to leave a straight scratch in the well and serum-free medium was added for incubation. Photographs of the wound were taken at 0 h and 24 h, and cell migration was assessed using the formula: wound healing rate (%) = (Wound width_0 h_−Wound width_24 h_)/Wound width_0 h_.

The cell suspension containing about 2 × 10^4^ cells starved overnight (200 μL) was seeded into the apical chamber, which was coated with 50 μL of 250 μg/mL Matrigel. The basolateral chamber was supplemented with 800 μL of RPMI-1640 medium (R7513, Sigma-Aldrich Chemical Company, St Louis, MO, USA) and 20% FBS. After 36 h of incubation, the cells were fixed with 4% paraformaldehyde for 30 min and stained with crystal violet staining solution for 15 min. The number of invaded cells was counted with a light microscope.

### Apoptosis analysis

Cells were washed with pre-cooled PBS and resuspended in 300 μL of binding buffer diluted with PBS. After 10 min of incubation, 5 μL of fluorescein isothiocyanate (FITC) Annexin V was added, followed by the incubation with 10 μL of propidium iodide (PI, ST511, Beyotime) for 5 min, and apoptosis was detected by flow cytometry within 1 h.

### RNA extraction and qPCR

Total RNA was isolated from A549 and HCC827 cells using TRIzol reagent, and the integrity of total RNA was determined on a 1% agarose gel. RNA was reverse transcribed to cDNA with HiScript III RT SuperMix (R323-01, NanJing Vazyme Biotech Co., Ltd, Nanjing, China). cDNA was PCR amplified with ChamQ Universal SYBR qPCR Master Mix (Q711-02, Vazyme) according to the manufacturer’s instructions. The final results were normalized to β-actin expression. For the analysis of amplification results, relative fold changes were calculated using the 2^−ΔΔCt^ method. The sequences of primers were as follows: KCTD9: forward primer: 5′-GCCGCTGTAATCTTGCACATGC-3′, reverse primer: 5′-CAGTTTCAGGGATGCTCCTTCTG-3′; TOP2A: forward primer: 5′-GTGGCAAGGATTCTGCTAGTCC-3′, reverse primer: 5′-ACCATTCAGGCTCAACACGCTG-3′; PD-L1: forward primer: 5′-TGCCGACTACAAGCGAATTACTG-3′, reverse primer: 5′-CTGCTTGTCCAGATGACTTCGG-3′; β-actin forward primer: 5′-CACCATTGGCAATGAGCGGTTC-3′, reverse primer: 5′-AGGTCTTTGCGGATGTCCACGT-3′.

### Western blot assay

Total proteins were isolated from the cells using a radioimmunoprecipitation assay buffer, and protein concentration was determined using the BCA Protein Concentration Assay Kit (P0012S, Beyotime). Protein samples were separated by SDS-PAGE, transferred to PVDF membranes, and blocked with 5% nonfat milk. The membranes were immunoblotted with antibodies to KCTD9 (1:10,000, ab180937, Abcam, Cambridge, UK), PD-L1 (1:1000, ab213524, Abcam), TOP2A (1:10,000, ab52934, Abcam), Ubiquitin (1:10,000, ab134953, Abcam), and GAPDH (D16H11) XP^®^ Rabbit mAb (1:1000, #5174, Cell Signaling Technologies, Beverly, MA, USA) at 4 °C overnight. The next day, the membranes were incubated in freshly prepared specific secondary antibody goat anti-rabbit IgG H&L (HRP) (1:2000, ab205718, Abcam) at room temperature for 2 h. Finally, the ECL kit was used for detection. Densitometric analysis of each band was performed using the Image J software.

### Enzyme-linked immunosorbent assay (ELISA)

The levels of tumor necrosis factor α (TNF-α), interferon-gamma (IFN-γ), CXCL10, and CXCL9 in the co-culture system were detected according to the instructions of Human TNF-α ELISA Kit (D711045, Shanghai Sangon Biological Engineering Technology & Services Co., Ltd., Shanghai, China), Human IFN-γ ELISA Kit (D711044, Sangon), Human CXCL10/IP-10 ELISA Kit (PC208, Beyotime), and MIG/CXCL9 Human ELISA Kit (EHCXCL9, Thermo Fisher).

The levels of TNF-α, IFN-γ, CXCL10, and CXCL9 in mouse xenograft tumors were detected according to the instructions of Mouse TNF-α ELISA Kit (PT512, Beyotime), Mouse IFN-γ ELISA Kit (PI508, Beyotime), Mouse CXCL10/IP-10 ELISA Kit (PC206, Beyotime), and MIG/CXCL9 Mouse ELISA Kit (EMCXCL9, Thermo Fisher).

### CD8^+^ T cell-mediated tumor cell killing assay

Isolated contaminant-free human CD8^+^ T cells were transferred to RPMI-1640 medium containing 10% FBS, 1% penicillin/streptomycin, and 1X MEM non-essential smino scid solution (11,140,050, Gibco, Carlsbad, CA, USA). Treatment with 1 mM sodium pyruvate (P2256, Merck KGaA, Darmstadt, Germany), 10 mM HEPES (H4034, Merck KGaA), 20 ng/mL human IL-2 recombinant protein (200–02-1MG, Gibco), and CD3/CD28 T cell activator (11161D, Gibco) was performed for 2 weeks. Activated CD8^+^ T cells were then co-cultured with A549 and HCC827 cells at a ratio of 3:1 for 48 h. After the removal of dead LUAD cells and the T cells from the medium, the live LUAD cells were stained with crystal violet and measured at 570 nm [35].

### In vitro co-culture system of mouse CD8 + T cells and A549 cells

The isolated mouse CD8^+^ T cells were activated as described above and then co-cultured with A549 cells in a 3:1 ratio for 48 h. A549 cells were treated with the PD-L1-neutralizing antibody MAb anti-human PD-L1 (BE0285, BioXCell, Shenzhen, Guangdong, China), and the control treatment was performed with MAb mouse IgG2b isotype control (BE0086, BioXCell). Samples were diluted according to the instructions and analyzed using Granzyme B Mouse ELISA Kit (88–8022-88, Invitrogen Inc., Carlsbad, CA, USA) and mouse Perforin ELISA Kit (NBP3-00452, Novus Biological Inc., Littleton, CO, USA).

### Co-immunoprecipitation (co-IP)

To detect the interaction between TOP2A and KCTD9, the A549 and HCC827 cells were lysed in 500 μL of co-IP buffer, and the solution was centrifuged for 30 min. The supernatant was immunoprecipitated with protein magnetic beads, which were incubated with the antibody against TOP2A (1:200, ab12318, Abcam). After overnight incubation at 4 °C, the protein magnetic beads were washed three times with co-IP buffer. SDS sample buffer was added to the protein magnetic beads, and the immunoprecipitates were subjected to western blot.

### In vivo tumor growth

All animal experiments were approved by the Animal Research Ethics Committee of the First Affiliated Hospital of Harbin Medical University. Six-week-old C57BL/6N mice (Vital River) were divided into four groups (n = 8): the control, CSE, CSE + sh-NC, and CSE + sh-KCTD9 groups. All mice were housed under specific pathogen-free conditions and provided with water and food. A549 cells (2 × 10^6^) with or without infection were injected subcutaneously into the right abdomen of mice. For CSE treatment, the mice were intravenously injected with 12.5 mL/kg of CSE per day. When the tumors became palpable, the volume of the tumors was measured weekly for three weeks according to the formula: volume = length × width^2^ × 0.5. After 3 weeks, the mice were euthanized by intraperitoneal injection of 150 mg/kg of sodium pentobarbital, and the lungs were weighed. Tumor tissues were dissected, fixed with 4% phosphate-buffered neutral formalin, and then embedded in paraffin for subsequent analysis.

### Immunofluorescence analysis

Paraffin-embedded sections of mouse tissues (4 μM) were baked at 60 °C for 120 min and deparaffinized. Antigen retrieval was conducted using EDTA buffer (pH = 8.0), and samples were blocked in 3% BSA containing 0.25% Triton X-100 for 1 h. The sections were incubated with CD8 Monoclonal Antibody (1:20, MA1-145, Thermo Fisher) overnight at 4 °C and with goat anti-rat IgG (H + L) Cross-Adsorbed Secondary Antibody, DyLight™ 488 (1:500, SA5-10,018, Thermo Fisher) for 2 h in the dark. After each staining, antigens were retrieved once in EDTA buffer (pH = 8.0) in a microwave oven. The washing in PBS-0.25% Triton X-100, the sections were incubated with DAPI solution for 10 min at room temperature, and the images were captured by fluorescence microscopy.

### TUNEL assay

Apoptosis in the tissue sections was measured according to the instructions of the TUNEL Apoptosis Detection Kit (Chromogenic Assay) (C1091, Beyotime). Tissue sections were deparaffinized with xylene for 5–10 min, treated with 20 μg/mL Proteinase K (ST532, Beyotime), and washed with Immunostaining Wash (P0106, Beyotime) at 20–37 °C for 15–30 min. The sections were incubated in 3% hydrogen peroxide solution prepared in PBS for 20 min at room temperature to inactivate endogenous peroxidase in the sections and treated with 50 μL of biotin labeling solution for 60 min at 37 °C in the dark. After incubation with 0.1–0.3 mL of labeling reaction termination solution for 10 min and 50 μL Streptavidin-HRP working solution for 30 min (both at room temperature), the sections were developed using 0.2–0.5 mL of DAB color development solution for 5–30 min at room temperature. The apoptotic cells were observed under a light microscope.

### Statistics

GraphPad Prism 9.5.0 (GraphPad, San Diego, CA, USA) was used for statistical analyses. All data were calculated as mean ± standard deviation (SD) for at least three independent experiments. Statistical analysis of multiple group comparisons was performed by one-way or two-way analysis of variance (ANOVA). A probability of 0.05 or less was considered statistically significant.

## Data Availability

The data that support the findings of this study are available from the corresponding author upon reasonable request.

## Abbreviations

LUAD: Lung adenocarcinoma
CSE: Coix seed extract
sh: Short hairpin RNA
NC: Negative control
OD: Optical density
PBS: Phosphate buffered saline
FITC: Fluorescein isothiocyanate
ELISA: Enzyme-linked immunosorbent assay
co-IP: Co-immunoprecipitation
